# Application of Global Warming Potential Star (GWP*) Values to the AUSNUT 2011-13 Food Composition Database: Creation of the GWP*-AUSNUT 2011-13 Database

**DOI:** 10.3390/nu17030464

**Published:** 2025-01-27

**Authors:** Elizabeth Neale, Troy Balvert, Hannah Crinnion, Joel Craddock, Kelly Lambert, Karen Charlton

**Affiliations:** School of Medicine, Health and Indigenous Sciences, Faculty of Science, Medicine and Health, University of Wollongong, Wollongong, NSW 2522, Australia; tab577@uowmail.edu.au (T.B.); hc102@uowmail.edu.au (H.C.); jcraddock@uow.edu.au (J.C.); klambert@uow.edu.au (K.L.); karenc@uow.edu.au (K.C.)

**Keywords:** climate change, global warming potential star, sustainability, AUSNUT 2011-13, food composition database

## Abstract

Background/Objectives: The Global Warming Potential Star (GWP*) refers to the amount of carbon dioxide equivalents produced by food items, with values available for *n* = 232 Australian food products. The aim of this study was to apply GWP* values to the AUSNUT 2011-13 food composition database to facilitate the calculation of the climate footprint of Australian dietary data. Methods: To create the GWP*-AUSNUT 2011-13 database, all *n* = 5740 food and beverage items in AUSNUT 2011-13 were reviewed and GWP* values applied or calculated via a systematic approach. Direct or approximate matches to a single GWP* value were prioritised. GWP* values were then calculated for composite foods with multiple ingredients. Finally, GWP* values were approximated based on food group, adjusted using other GWP* values, or foods were excluded if no appropriate match could be found. Results: A total of *n* = 5502 (95.85%) AUSNUT 2011-13 foods were matched to a GWP* value, with the majority requiring calculation based on multiple ingredients. Mean ± standard deviation GWP* values ranged from 0.18 ± 0.12 kg CO_2_e/kg (‘Dairy and meat substitutes’) to 5.63 ± 7.55 kg CO_2_e/kg (‘Meat, poultry and game products and dishes’). Conclusions: The GWP*-AUSNUT 2011-13 database can be applied to Australian dietary data to identify the climate footprint of different dietary patterns or to provide insight into dietary changes required to decrease greenhouse gas emissions. Future research is now required to develop new GWP* values for a broader range of foods and to update this database when new Australian food composition databases are released.

## 1. Introduction

Climate change is a substantial threat to humanity, with the Intergovernmental Panel on Climate Change recommending that immediate action is required to limit increasing global temperatures [1]. The food supply is a large contributor to greenhouse gas emissions, with food systems contributing approximately 34% of total emissions globally [2]. Of these emissions, approximately 40% are associated with primary production, 32% with land use or land use change activities, 18% with the supply chain (including food processing, transport, packaging, and retail), and 12% with post-retail stages such as cooking and waste [2]. As a result, changes to both food production and dietary patterns are required to reduce greenhouse gas emissions from the food supply. In order to assess the impact of current diets and plan strategies to reduce emissions, quantification of the climate impact of a range of foods is required.

A number of methods exist for quantifying the climate impact of foods. Dietary greenhouse gas emissions have most commonly been studied using the 100-year Global Warming Potential (GWP100) [3], which reports the global warming potential over a 100-year time horizon. In contrast to the GWP100, the Global Warming Potential Star (GWP*) metric evaluates the climate impact by specifically including cumulative impacts from CO_2_ and the additional contribution of short-lived gases such as methane [4,5,6]. Given the role of the food system in producing methane, the GWP* metric provides valuable insights into the potential climate impact of foods and dietary patterns [7]. At present, GWP* values are available for 232 Australian foods [7].

Previous studies have applied GWP* values to specific diets, including healthy diets, typical Australian diets, and therapeutic diets [8,9,10], as well as national survey data [7]. However, in order to quantify the climate impact of Australian diets from all food sources, GWP* values must be applied to all foods in AUSNUT 2011-13 [11], the most current Australian food composition database.

The aim of this study was to, therefore, systematically apply GWP* values to foods and beverages in AUSNUT 2011-13 to facilitate the calculation of the climate footprint of Australian dietary data.

## 2. Materials and Methods

**Figure 1:**
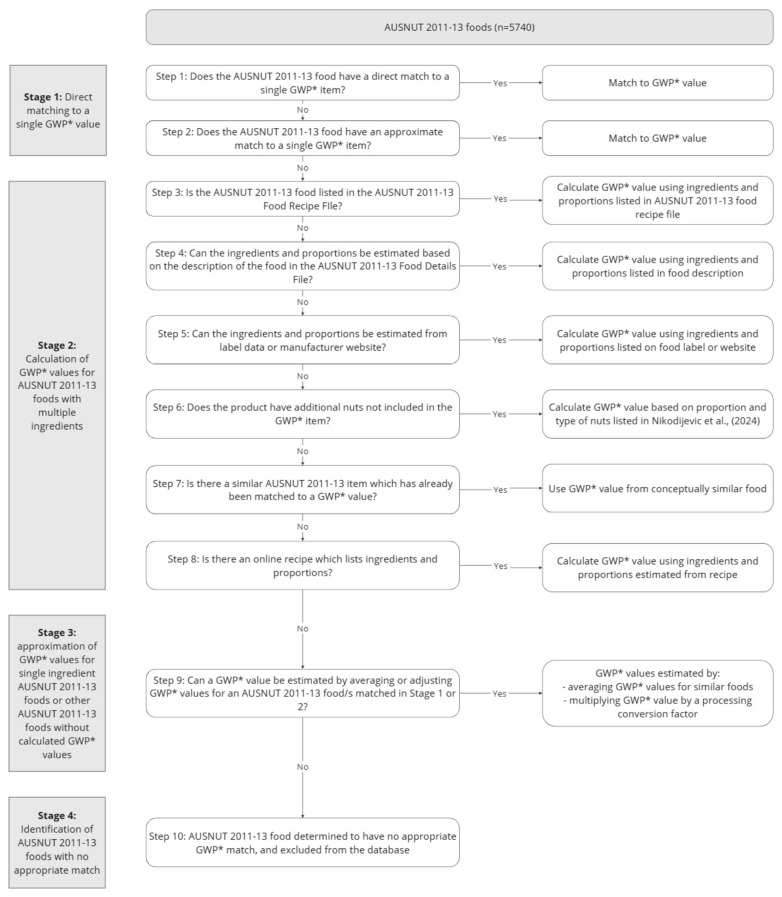
Process of applying GWP* values to AUSNUT 2011-13 foods [14].

To create the GWP*-AUSNUT 2011-13 database, all *n* = 5740 food and beverage items in AUSNUT 2011-13 were reviewed, and GWP* values were applied or calculated via a systematic approach—shown in Figure 1 and outlined in further detail below. The process of matching AUSNUT 2011-13 foods to GWP* values involved data from a range of sources, including the AUSNUT 2011-13 food details file (which contains the food name, description, and list of included and excluded items, including brand names) [12], the AUSNUT 2011-13 food recipe file (which contains the ingredients and their proportions for composite foods with multiple ingredients within AUSNUT 2011-13) [13], the AUSNUT 2011-13 food nutrient database (which contains the nutrient data for all AUSNUT 2011-13 foods and beverages) [11], a published database of nut content of AUSNUT 2011-13 foods and beverages [14], as well as label data for commercially available products and online recipes. The climate impact values were limited to the *n* = 232 GWP* values determined by Ridoutt et al. [7], with the exception of salt. While Ridoutt et al. [7] do not include a GWP* value for salt, due to the ubiquitous nature of salt within the food supply, a value of 0.06 kg CO_2_e/kg was obtained from CarbonCloud.com [15] and used in the creation of the present database.

The process of applying GWP* values to AUSNUT 2011-13 foods was completed in duplicate by two researchers (one an Advanced Accredited Practising Dietitian). Discrepancies were initially discussed between researchers responsible for matching foods, with outstanding issues discussed with the remaining members of the research team (K.L and K.C) until consensus was reached. Values for the final GWP*-AUSNUT 2011-13 database were presented to two decimal places to align with the reporting used for most nutrients in the AUSNUT 2011-13 food nutrient database.

### 2.1. Stage 1: Direct Matching to a Single GWP* Value

Where possible, direct matching of AUSNUT 2011-13 foods to a single GWP* value was prioritised. Matching of AUSNUT 2011-13 foods was based on conceptual similarities informed by ingredients and cooking methods; for example, the AUSNUT 2011-13 food ‘*Beef, mince, <5% fat, raw*’ was matched to the GWP* item ‘Beef meat’, with a GWP* value of 16.68 kg CO_2_e/kg (Step 1). Where a direct match was not possible, the closest approximate match to a single GWP* value was made (Step 2). For example, while GWP* values refer to uncooked foods, the AUSNUT 2011-13 food ‘*Beef, mince, <5% fat, baked, roasted, fried or stir-fried, grilled or BBQ’d, no added fat*’ is cooked with no additional fat or other ingredients, and therefore the GWP* value for ‘Beef meat’ was deemed to be the closest match. Where the AUSNUT 2011-13 food was listed in the AUSNUT 2011-13 food recipe file, the recipe file was checked to determine if any additional ingredients were present that would influence the GWP* value used.

### 2.2. Stage 2: Calculation of GWP* Values for AUSNUT 2011-13 Foods with Multiple Ingredients

While some of the published GWP* values refer to composite foods with multiple ingredients (e.g., ‘Muffin, cake-style, berry, commercial, uniced’), most AUSNUT 2011-13 foods with more than one ingredient could not be matched to a single GWP* value. Where possible, GWP* values for composite foods were calculated using the AUSNUT 2011-13 food recipe file (Step 3). In order to do so, the proportion of each ingredient in the composite food was first calculated. This proportion was then multiplied by each ingredient’s GWP*, and these values were then summed to calculate a total GWP* value for the composite food. An example of this calculation (for a composite food with three ingredients) is shown in Equation (1). Where any of the ingredients were determined not to have a GWP* match (as outlined below in Stage 4), these ingredients and their weight were excluded from the recipe calculation to avoid inaccuracies in the total GWP* calculation.

(1) $$\begin{array}{c} Total\;GWP*\;of\;composite\;food\\ \;\;\;\;\;\;\;\;\;\;\;\;\;\;\;\;\;\;\;\;\;\;\;\;\;\;\;=(\left[weight\;of\;ingredient\;1÷total\;weight\;of\;composite\;food\right]\times GWP*\;value\;of\;ingredient\;1)\\ \;\;\;\;\;\;\;\;\;\;\;\;\;\;\;\;\;\;\;\;\;\;\;\;\;\;\;+(\left[weight\;of\;ingredient\;2÷total\;weight\;of\;composite\;food\right]\times GWP*\;value\;of\;ingredient\;2)\\ \;\;\;\;\;\;\;\;\;\;\;\;\;\;\;\;\;\;\;\;\;\;\;\;\;\;\;+(\left[weight\;of\;ingredient\;3÷total\;weight\;of\;composite\;food\right]\times GWP*\;value\;of\;ingredient\;3) \end{array}$$

Equation (1) calculates the total GWP* for composite foods (using a composite food with three ingredients as an example).

For composite foods that were not listed in the AUSNUT 2011-13 food recipe file, GWP* values were calculated based on the description of the food (Step 4), label or manufacturer information (Step 5), or other published data (Step 6); substituted with values from a similar food (Step 7); or calculated based on online recipes (Step 8). This process is discussed in further detail below.

Where foods were not listed in the AUSNUT 2011-13 food recipe file, recipes were estimated using the description of the food in the AUSNUT 2011-13 Food Details File (Step 4); for example, the description of ‘*Tea, regular, black, brewed from leaf or teabags, plain, without milk’* states that this food contains 15 g of tea leaves to one litre of water. GWP* values were also calculated based on label or manufacturer data where these were available (Step 5), for example, for the AUSNUT 2011-13 food ‘*Peanut butter, smooth & crunchy, added sugar & salt*’. To do so, a sample of up to five products matching the AUSNUT 2011-13 food was identified from the two major Australian supermarkets. Where more than five eligible products existed, a sample of five products that reflected the variety of brands available was selected. The individual ingredients were then identified from the product labels, with the percentage of ingredients obtained from the label where possible, and the average percentage calculated across the sample of products. Proportions of the ingredients not listed as a percentage on the label were estimated based on their order in the ingredients list and the nutritional composition of the AUSNUT 2011-13 food. For AUSNUT 2011-13 foods that were prepared via the addition of liquids (for example, ‘*Barley, pearl, cooked in water, no added fat or salt’*), preparation instructions on the label were used to determine the proportion of liquid used. For fast food products, for example, ‘*Hamburger, white roll, beef patty, with cheese, lettuce, sauce, fast food chain*’, ingredients and proportions were based on information from manufacturer websites. After estimating ingredients and their proportions, the nutritional composition of the AUSNUT 2011-13 food (e.g., ‘*Peanut butter, smooth & crunchy, added sugar & salt*’) was checked against that of the estimated ingredients (e.g., peanuts, vegetable oil, sugar, salt) using Foodworks dietary analysis software (Version 10, Xyris Software, Brisbane, QLD, Australia, 2019), and proportions of ingredients were adjusted where required. The GWP* values of the individual ingredients were then multiplied by their proportion in the overall food and summed to obtain a total GWP* value for the product using the same process followed in Step 3.

Some composite AUSNUT 2011-13 food items without recipes were identified as being an approximate match to a GWP* value but contained additional components that could be quantified (for example, the AUSNUT 2011-13 food ‘*Breakfast cereal, flakes of corn, added nuts, added vitamins B1, B2, B3, C & folate, Fe & Zn*’ was an approximate match for the GWP* item ‘Cornflake’, but contained additional nuts). Given that the GWP* value of nuts differs from other plant foods, it was considered important to estimate the amount of nuts in these products. As a result, the proportion and types of nuts in these foods were determined using a published database [14] (Step 6), and the total GWP* value calculated using the same approach as other recipes.

For composite foods without an AUSNUT 2011-13 recipe, description, or product label, calculated GWP* values were obtained from conceptually similar foods where possible (for example, the calculated GWP* value for AUSNUT 2011-13 food ‘*Pizza, supreme, thin base, takeaway style*’ was used for ‘*Pizza, supreme, thin base, fast food chain*’) (Step 7). Finally, ingredients and proportions were estimated based on online recipes (for example, the GWP* value for ‘*Ice cream, Cassata-style dessert*’ was calculated based on the ingredients and amounts listed in an online recipe) (Step 8). Due to the variation in these recipes, however, use of this approach was limited where possible.

### 2.3. Stage 3: Approximation of GWP* Values for Other AUSNUT 2011-13 Foods Without Calculated GWP* Values

In the case of AUSNUT 2011-13 foods that did not have a direct or approximate match to a single GWP* value (Stage 1) or a calculated GWP* value based on multiple ingredients (Stage 2), GWP* values were approximated based on other foods in the same food group or adjusted based on other GWP* values (Step 8).

For AUSNUT 2011-13 foods that could not be matched in Stages 1 or 2, an average of GWP* values for similar foods was calculated. Where possible, the selection of similar foods was predominantly based on the AUSNUT 2011-13 food classification system; for example, as there was no GWP* match for ‘*Nut, brazil, with or without skin, raw, unsalted*’, an average of all GWP* items aligning with foods in the AUSNUT 2011-13 minor food group ‘Other nuts and nut products and dishes’ (‘hazelnut’, ‘cashew’, ‘walnut’, ‘pistachio’, ‘almond’) was calculated.

Due to the variation in GWP* values for processed foods such as canned fruits and vegetables compared to their fresh counterparts, an approximation of a GWP* value for processed foods was required where this did not exist. To do so, a processing conversion factor for fruits and for vegetables was approximated by dividing the GWP* values for the processed fruit or vegetable (e.g., ‘Pear, processed’) by its fresh counterpart (e.g., ‘Pear, fresh’) and then calculating an average processing factor for fruit and vegetables. This average conversion factor was then applied to approximate the GWP* value of the processed food.

### 2.4. Step 4: Identification of AUSNUT 2011-13 Foods with No Appropriate Match

Finally, AUSNUT 2011-13 foods that did not have an appropriate GWP* match were identified. These included single-ingredient foods that were considered to differ substantially from the GWP* items, for example, wild-caught meats such as ‘*Echidna, wild caught, flesh, raw*’, where it was concluded that the climate impact of these foods would not be comparable to conventionally farmed meats. Other types of AUSNUT 2011-13 foods with no appropriate match included composite AUSNUT 2011-13 foods whose constituent ingredients did not have appropriate GWP* matches (e.g., ‘*Meal replacement powder, coffee flavour*’), and where there were no other similar foods with a GWP* match (e.g., ‘*Cheese, soy*’). These products were, therefore, not assigned a GWP* value and were excluded from the database.

### 2.5. Data Analysis

After the creation of the GWP*-AUSNUT 2011-13 database, the percentage of AUSNUT 2011-13 foods that could be matched to GWP* values was calculated. In addition, the proportion directly matched to GWP* values (Stage 1), calculated based on multiple ingredients (Stage 2), or approximated (Stage 3) was calculated. The mean and standard deviation GWP* values were also calculated for AUSNUT 2011-13 major and sub-major food groups.

## 3. Results

Of the *n* = 5740 foods and beverages in the AUSNUT 2011-13 database, *n* = 5502 (95.85%) were matched to a GWP* value, with the remaining foods determined to not have an appropriate GWP* match. More than half of AUSNUT 2011-13 foods were matched to GWP* values at Stage 2, with the majority of these based on the ingredients and proportions listed in the AUSNUT 2011-13 food recipe file (Table 1).

The mean and standard deviation GWP* (kg CO_2_e/kg) is shown in Table 2 according to the AUSNUT 2011-13 major food group. The food group with the highest mean GWP* value was ‘Meat, poultry and game products and dishes’ (5.63 + 7.55 kg CO_2_e/kg), while the food group with the lowest mean GWP* value was ‘Dairy & meat substitutes’ (0.18 + 0.12 kg CO_2_e/kg).

## 4. Discussion

This research outlines the application of GWP* values to an Australian food composition database via a systematic approach. The resultant GWP*-AUSNUT 2011-13 database may now be applied to dietary data to calculate the climate impact of diets consumed in Australia. Given the considerable impact of the food supply on climate change [2], this database provides valuable insights for quantifying the effects of current dietary patterns and for planning diets with reduced climate impact.

The creation of the GWP*-AUSNUT 2011-13 database required a systematic approach based on conceptual similarities and understanding of food composition. A hierarchical approach was used, prioritising direct and approximate matches to existing GWP* items, followed by using published data on food components for composite foods with multiple ingredients [13]. This approach aligns with those previously used when expanding the AUSNUT 2011-13 food composition database to include data on specific food components, such as nuts [14], whole grains [17], and plant-based foods [18]. While direct and approximate matches to existing GWP* items were prioritised where possible, more than half of the foods in AUSNUT 2011-13 had GWP* values calculated based on multiple ingredients. This pattern reflects the predominance of composite foods with more than one ingredient in AUSNUT 2011-13, in comparison to GWP* values, which were most commonly available for single-ingredient foods such as meats, fruits, and vegetables. While the use of the published AUSNUT 2011-13 food recipe file for the majority of these calculations increases the transparency and replicability of the approach used in the current study, it is possible that the combined GWP* values of the constituent ingredients may differ from that of the composite food overall. There is, therefore, a need for further determination of GWP* values for multi-ingredient foods via life cycle analysis.

A key challenge when creating the GWP*-AUSNUT 2011-13 database was the limited number of foods, with GWP* data available for only 232 foods [7]. While direct and approximate matches to GWP* items were prioritised where possible (and these values were subsequently used in calculations of composite foods with more than one ingredient), a substantial proportion of AUSNUT 2011-13 foods and beverages could not be matched to GWP* values in Stages 1 or 2. These included items such as specific varieties of fruits and vegetables and composite foods with ingredients that could not be matched. While GWP* values for these products were approximated where possible (for example, by taking the average of a food group), approximately 4% of AUSNUT 2011-13 foods were deemed to be unable to be matched to a GWP* value and were therefore excluded from the database. These exclusions may result in an underestimation of the climate impact of overall diets, highlighting the need for additional life cycle analysis data on a broader range of foods. In particular, there is a need for further data on meat alternatives such as vegetarian sausages and soy cheese, as well as protein powders and meal replacements. Given the increasing popularity of products such as meat alternatives in the Australian diet [19,20], and the limited options for appropriate substitution with existing GWP* items, these products should be prioritised for life cycle analysis. In addition, other items that should be considered for the calculation of GWP* values include foods frequently used as ingredients in composite foods (such as dried herbs), as well as wild-caught meats whose climate impact may differ substantially from conventionally farmed meats [21,22].

Development of the GWP*-AUSNUT 2011-13 database illustrated the differences in the climate impact of different food groups. The food groups with the highest mean GWP* values were ‘Meat, poultry and game products and dishes’ followed by ‘Fish and seafood products and dishes’. These patterns reflect the characteristics of these food groups, for example, methane production, particularly for red meat such as beef [23]. It should, however, be noted that GWP* values within each food category may vary substantially between foods. For example, the GWP* value of lamb is much lower than that of beef, pork, or chicken due to the nature of Australian sheep husbandry resulting in lower overall and ongoing declines in methane emissions and a negative GWP* value [23]. In comparison, the food group with the lowest GWP* values were ‘Dairy & meat substitutes’ and ’Legume and pulse products and dishes’, reflecting the lower greenhouse gas emissions associated with their production [24]. Values for the ‘Dairy & meat substitutes’ should be interpreted with caution, however, due to the lack of matching GWP* items resulting in some foods being excluded from the database (e.g., ‘*cheese, soy*’) or substituted with GWP* items that may differ from the AUSNUT 2011-13 food (for example, the AUSNUT 2011-13 food ‘*Sausage, vegetarian style, raw*’ was matched to ‘tofu’ as the closest match). Taken together, however, these findings across food groups align with dietary recommendations such as the EAT-Lancet diet [25], which recommends a diet lower in red meat and higher in plant-based alternatives such as legumes for both human health and environmental sustainability.

Previous studies have applied GWP* values to Australian dietary data. Clay et al. [8] and O’Brien et al. [10] calculated the climate impact of a range of diets, including therapeutic diets (for chronic kidney disease, coeliac disease, and type 2 diabetes), the current Australian diet, and the EAT-Lancet Planetary Health Diet, while Cobben et al. [9] calculated GWP* values for a typical Australian diet and a heart-healthy diet. These studies used a similar approach to applying GWP* values to the present study, including calculating GWP* values based on the proportions of individual ingredients for composite foods. In comparison to the present study, however, these previous studies only calculated GWP* values for a subset of Australian foods. The comprehensive application of GWP* values to all AUSNUT 2011-13 foods in the current study means that these values can now be applied to all dietary data collected using AUSNUT 2011-13, allowing for transparent and consistent comparison of climate impacts between diets and studies. After compiling the GWP* values, Ridoutt et al. [7] applied these values to data from the 2011-13 Australian Health Survey in order to calculate the climate impact of the Australian diet. However, the methods used to apply these values to Australian foods are not publicly available, making comparisons between this and the current approach challenging. It should be noted, however, that differences in the approaches used between Ridoutt et al. [7] and the present study may result in differences in the total GWP* calculated if applied to the same dataset.

Estimation of diet-related greenhouse gas emissions using other methods based on life cycle analysis has also been conducted in regions other than Australia; for example, the United States [26], the United Kingdom [27,28], Japan [29], the Netherlands [30], Sweden [31], Denmark [32], and France [33], as well as analysis of data from multiple European countries [34]. While this involved applying estimations for greenhouse gas emissions to dietary data, many studies calculated diet-related emissions based on broad food groups or through dietary modelling using a subset of commonly consumed foods, with few studies reporting the process used to systematically apply values to all foods within a national food composition database. One study that reported a detailed approach to applying estimations of greenhouse gas emissions to a food composition database was that of Sugimoto et al. [29], who developed a greenhouse gas emissions database for Japanese foods using three different estimation methods. Of note, the process followed by Sugimoto et al. to assign values to foods had similarities with the approach used in the present study. This included a staged approach that prioritised direct or approximate food matches, substituted values for similar foods or foods in the same food group, and calculated values for composite foods using a recipe approach. While these processes do require extrapolation or substitution of values for foods without greenhouse gas emission data, assigning values to all (or close to all) individual foods within a food composition database allows for the comprehensive analysis of dietary data collected using these databases.

Released in 2014, AUSNUT 2011-13 [11] is currently the most recent survey-specific food composition database in Australia. Ongoing changes in the food supply and food consumption patterns since AUSNUT 2011-13 was developed must be considered when interpreting the results of the present study. For example, sales of ultra-processed foods in many countries, including Australia, are high and are continuing to increase [35]. Ultra-processed foods are known to have substantial environmental impacts, accounting for between 27–35% of dietary greenhouse gases in Australia, a finding that is in part driven by the high consumption of these foods [36]. Given the increasing consumption of such foods in Australia, and their environmental impact, future updates to the GWP*-AUSNUT 2011-13 database to align with new versions of AUSNUT should pay particular attention to ultra-processed foods, including considering additional life cycle analysis of these foods. In addition, other significant changes to the Australian food supply and consumption patterns since the release of AUSNUT 2011-13, for example, the impact of the COVID-19 pandemic [37,38], may influence the applicability of these results to the current food supply. It should be noted that a new version of AUSNUT is expected to be released in 2025. When released, the present GWP*-AUSNUT 2011-13 database should be updated to the most current food composition database using the methods outlined in the current study.

The creation of the GWP*-AUSNUT 2011-13 database was strengthened by its systematic approach, with the process of applying GWP* values to AUSNUT 2011-13 foods completed in duplicate and discussed with a broader team of experienced dietitians. In addition, information from published data sources, such as descriptions of foods in the AUSNUT 2011-13 food details file [12] or from the AUSNUT 2011-13 food recipe file [13], were prioritised where possible, increasing the transparency and replicability of the approach used. However, there were a number of limitations to this study. As a result of the limited number of foods with published GWP* values, substitutions with other food items or approximations of GWP* calculations were required. While these were based on the study author’s (E.N.) professional judgement as an Advanced Accredited Practising Dietitian and determined by consensus with the research team, they may result in inaccuracies when calculating the climate impact of an overall diet. In addition, while these potentially inaccurate foods may be isolated when applying the database, many were also used as ingredients in composite foods calculated in Stage 2, meaning they may have had widespread implications throughout the database. When calculating GWP* values for composite foods, ingredients excluded from the GWP*-AUSNUT 2011-13 database were not included in the composite food calculation. While this was determined to have minimal impact on results as most excluded foods made up a very small proportion of the composite food recipe (for example, dried herbs), in some cases, such as protein powders (excluded from the database due to a lack of appropriate match) prepared with water, the only eligible ingredient was water (with a GWP* value of 0 kg/CO_2_ kg). While this resulted in an underestimation of the GWP* values of these foods, it was considered appropriate in order to be consistent with the overall approach to calculating recipes and was found to impact only a limited number of foods. In addition, the GWP* values used in this study do not include emissions from packaging, kitchen storage, or meal preparation [7], resulting in an underestimation of the total CO_2_e produced. While these components are not the largest contributors to overall emissions from the food system, they are nonetheless considerable. For instance, it has been estimated that approximately 5.4% of total food system emissions arise from packaging, while domestic food preparation and waste (including solid waste disposal and wastewater) account for approximately 12% of emissions [2]. Finally, the current version of AUSNUT relates to the Australian Health Survey conducted in 2011–2012 [39]. As a result, data collected from current product labels is likely to differ from the original sources available in AUSNUT 2011-13.

## 5. Conclusions

The GWP*-AUSNUT 2011-13 database was created through application of GWP* values to the AUSNUT 2011-13 food composition database via a systematic process. This database will allow for the estimation of the climate impact from dietary data collected in Australia, both retrospectively and in future studies, to identify the climate footprint of different dietary patterns or to provide insight into dietary changes required to decrease greenhouse gas emissions. Future research is now required to develop new GWP* values for a broader range of foods and to update this database when new Australian food composition databases are released.

## Figures and Tables

**Table 1:** Number of AUSNUT 2011-13 foods matched at each stage and step.

Stage and Step of Matching Process	Number (%) of AUSNUT 2011-13 Foods Matched *
Stage 1: Direct matching to a single GWP* value	*2022 (35.23)*
- Direct match to GWP* item	883 (15.38)
- Approximate match to GWP* item	1139 (19.84)
Stage 2: Calculation of GWP* values for AUSNUT 2011-13 foods with multiple ingredients	*3136 (54.63)*
- Calculated using AUSNUT 2011-13 food recipe file	2709 (47.20)
- Calculated based on a description of ingredient quantities in AUSNUT 2011-13 food details file	31 (0.54)
- Calculated based on label or manufacturer data	104 (1.81)
- Calculated using proportion and type of nuts based on Nikodijevic et al. [14]	24 (0.42)
- Based on calculated GWP* value for similar food	258 (4.49)
- Calculated based on online recipe	10 (0.17)
Stage 3: Approximation of GWP* values for other AUSNUT 2011-13 foods without calculated GWP* values	*344 (5.99)*
- Approximated using the average of food group	326 (5.68)
- Processing factor applied	18 (0.31)
*Step 4: Identification of AUSNUT 2011-13 foods with no appropriate match*	*238 (4.15)*

* Percentages may not sum up to 100% due to rounding.

**Table 2:** Mean and standard deviation GWP* (kg CO_2_e/kg), by AUSNUT 2011-13 major food groups *.

AUSNUT 2011-13 Major Food Group	GWP* (kg CO_2_e/kg) (Mean ± SD)
Non-alcoholic beverages	0.87 ± 1.72
Cereals and cereal products	0.58 ± 0.41
Cereal-based products and dishes	1.87 ± 1.50
Fats and oils	2.99 ± 2.44
Fish and seafood products and dishes	4.35 ± 4.07
Fruit products and dishes	0.72 ± 0.46
Egg products and dishes	1.76 ± 0.49
Meat, poultry, and game products and dishes	5.63 ± 7.55
Milk products and dishes	2.92 ± 2.16
Dairy and meat substitutes	0.18 ± 0.12
Soup	0.70 ± 0.77
Seed and nut products and dishes	1.94 ± 0.98
Savoury sauces and condiments	1.30 ± 1.37
Vegetable products and dishes	0.66 ± 0.61
Legume and pulse products and dishes	0.28 ± 0.13
Snack foods	2.68 ± 1.16
Sugar products and dishes	1.24 ± 1.20
Confectionery and cereal/nut/fruit/seed bars	2.31 ± 1.23
Alcoholic beverages	0.54 ± 0.35
Special dietary foods	0.45 ± 0.47
Miscellaneous	0.36 ± 0.78
Infant formulae and foods	0.70 ± 0.56
Reptiles, amphibia, and insects †	-

* Details of the AUSNUT 2011-13 major food groups are available from Food Standards Australia New Zealand [16]. † No foods in this food group could be matched to GWP* values.

## Data Availability

The datasets presented in this article are not readily available because the data are part of an ongoing study. Requests for further information regarding datasets should be directed to the corresponding author.

## References

[1] Intergovernmental Panel on Climate Change (2023). Climate Change 2022—Mitigation of Climate Change: Working Group III Contribution to the Sixth Assessment Report of the Intergovernmental Panel on Climate Change.

[2] Crippa M., Solazzo E., Guizzardi D., Monforti-Ferrario F., Tubiello F.N., Leip A. (2021). Food systems are responsible for a third of global anthropogenic GHG emissions. Nat. Food.

[3] Ridoutt B.G., Hendrie G.A., Noakes M. (2017). Dietary Strategies to Reduce Environmental Impact: A Critical Review of the Evidence Base. Adv. Nutr..

[4] Allen M.R., Shine K.P., Fuglestvedt J.S., Millar R.J., Cain M., Frame D.J., Macey A.H. (2018). A solution to the misrepresentations of CO_2_-equivalent emissions of short-lived climate pollutants under ambitious mitigation. NPJ Clim. Atmos. Sci..

[5] Cain M., Lynch J., Allen M.R., Fuglestvedt J.S., Frame D.J., Macey A.H. (2019). Improved calculation of warming-equivalent emissions for short-lived climate pollutants. NPJ Clim. Atmos. Sci..

[6] Lynch J., Cain M., Pierrehumbert R., Allen M. (2020). Demonstrating GWP*: A means of reporting warming-equivalent emissions that captures the contrasting impacts of short- and long-lived climate pollutants. Environ. Res. Lett..

[7] Ridoutt B., Baird D., Hendrie G.A. (2021). Diets within Environmental Limits: The Climate Impact of Current and Recommended Australian Diets. Nutrients.

[8] Clay N., Charlton K., Stefoska-Needham A., Heffernan E., Hassan H.I.C., Jiang X., Stanford J., Lambert K. (2023). What is the climate footprint of therapeutic diets for people with chronic kidney disease? Results from an Australian analysis. J. Hum. Nutr. Diet..

[9] Cobben R.E., Collins C.E., Charlton K.E., Bucher T., Stanford J. (2024). Sustainability and cost of typical and heart-healthy dietary patterns in Australia. Am. Heart J. Plus Cardiol. Res. Pract..

[10] O’Brien R., Cosier D., Lambert K. (2025). The climate footprint of the diabetic and gluten free diet in Australia. Dietetics.

[11] Food Standards Australia New Zealand (2014). AUSNUT 2011-13—Australian Food Composition Database.

[12] Food Standards Australia New Zealand (2014). AUSNUT 2011-13—Food Details File.

[13] Food Standards Australia New Zealand (2014). AUSNUT 2011-13—Food Recipe File.

[14] Nikodijevic C.J., Probst Y.C., Tan S.Y., Neale E.P. (2024). Metabolisable energy from nuts and patterns of nut consumption in the Australian population: A secondary analysis of the 2011–2012 National Nutrition and Physical Activity Survey. J. Hum. Nutr. Diet..

[15] Carbon Cloud Salt (NaCl). https://apps.carboncloud.com/climatehub/product-reports/id/5403842093.

[16] Food Standards Australia New Zealand Classification of Foods and Dietary Supplements. https://www.foodstandards.gov.au/science-data/food-composition-databases/ausnut/classificationofsupps.

[17] Galea L.M., Dalton S.M.C., Beck E.J., Cashman C.J., Probst Y.C. (2016). Update of a database for estimation of whole grain content of foods in Australia. J. Food Compos. Anal..

[18] Stanford J., McMahon S., Lambert K., Charlton K.E., Stefoska-Needham A. (2023). Expansion of an Australian food composition database to estimate plant and animal intakes. Br. J. Nutr..

[19] Brooker P.G., Hendrie G.A., Anastasiou K., Woodhouse R., Pham T., Colgrave M.L. (2022). Marketing strategies used for alternative protein products sold in Australian supermarkets in 2014, 2017, and 2021. Front. Nutr..

[20] Kalocsay K., Eassom S., King T., O’Neill S., Cogo K., Redmond M. (2024). 2023 State of the Industry: Australia’s Plant-Based Meat Industry.

[21] Johnson J.L., Zamzow B.K., Taylor N.T., Moran M.D. (2021). Reported U.S. wild game consumption and greenhouse gas emissions savings. Hum. Dimens. Wildl..

[22] Nunes A.V., Peres C.A., Constantino P.d.A.L., Fischer E., Nielsen M.R. (2021). Wild meat consumption in tropical forests spares a significant carbon footprint from the livestock production sector. Sci. Rep..

[23] Ridoutt B. (2021). Short communication: Climate impact of Australian livestock production assessed using the GWP* climate metric. Livest. Sci..

[24] Semba R.D., Ramsing R., Rahman N., Kraemer K., Bloem M.W. (2021). Legumes as a sustainable source of protein in human diets. Glob. Food Secur..

[25] Willett W., Rockström J., Loken B., Springmann M., Lang T., Vermeulen S., Garnett T., Tilman D., DeClerck F., Wood A. (2019). Food in the Anthropocene: The EAT–Lancet Commission on healthy diets from sustainable food systems. Lancet.

[26] Hitaj C., Rehkamp S., Canning P., Peters C.J. (2019). Greenhouse Gas Emissions in the United States Food System: Current and Healthy Diet Scenarios. Environ. Sci. Technol..

[27] Hoolohan C., Berners-Lee M., McKinstry-West J., Hewitt C. (2013). Mitigating the greenhouse gas emissions embodied in food through realistic consumer choices. Energy Policy.

[28] Green R., Milner J., Dangour A.D., Haines A., Chalabi Z., Markandya A., Spadaro J., Wilkinson P. (2015). The potential to reduce greenhouse gas emissions in the UK through healthy and realistic dietary change. Clim. Change.

[29] Sugimoto M., Murakami K., Asakura K., Masayasu S., Sasaki S. (2021). Diet-related greenhouse gas emissions and major food contributors among Japanese adults: Comparison of different calculation methods. Public Health Nutr..

[30] van de Kamp M.E., van Dooren C., Hollander A., Geurts M., Brink E.J., van Rossum C., Biesbroek S., de Valk E., Toxopeus I.B., Temme E.H. (2018). Healthy diets with reduced environmental impact?–The greenhouse gas emissions of various diets adhering to the Dutch food based dietary guidelines. Food Res. Int..

[31] Sjörs C., Raposo S.E., Sjölander A., Bälter O., Hedenus F., Bälter K. (2016). Diet-related greenhouse gas emissions assessed by a food frequency questionnaire and validated using 7-day weighed food records. Environ. Health.

[32] Saxe H., Larsen T.M., Mogensen L. (2013). The global warming potential of two healthy Nordic diets compared with the average Danish diet. Clim. Change.

[33] Masset G., Vieux F., Verger E.O., Soler L.-G., Touazi D., Darmon N. (2014). Reducing energy intake and energy density for a sustainable diet: A study based on self-selected diets in French adults. Am. J. Clin. Nutr..

[34] Hartikaiinen H., Pulkkinen H. (2016). Summary of the Chosen Methodologies and Practices to Produce GHGE-Estimates for an Average European Diet.

[35] Baker P., Machado P., Santos T., Sievert K., Backholer K., Hadjikakou M., Russell C., Huse O., Bell C., Scrinis G. (2020). Ultra-processed foods and the nutrition transition: Global, regional and national trends, food systems transformations and political economy drivers. Obes. Rev..

[36] Anastasiou K., Baker P., Hadjikakou M., Hendrie G.A., Lawrence M. (2022). A conceptual framework for understanding the environmental impacts of ultra-processed foods and implications for sustainable food systems. J. Clean. Prod..

[37] Godrich S.L., Macau F., Kent K., Lo J., Devine A. (2022). Food Supply Impacts and Solutions Associated with the COVID-19 Pandemic: A Regional Australian Case Study. Int. J. Environ. Res. Public Health.

[38] Louie S., Shi Y., Allman-Farinelli M. (2022). The effects of the COVID-19 pandemic on food security in Australia: A scoping review. Nutr. Diet..

[39] Australian Bureau of Statistics (2013). 4363.0.55.001—Australian Health Survey: Users’ Guide, 2011–2013.

